# Intensive management for moderate rheumatoid arthritis: a qualitative study of patients’ and practitioners’ views

**DOI:** 10.1186/s41927-019-0057-8

**Published:** 2019-03-28

**Authors:** Louise Prothero, Jackie Sturt, Savia de Souza, Heidi Lempp, on behalf of TITRATE Programme Investigators, Heidi Lempp, Jackie Sturt, Sofia Georgopoulou, Louise Prothero, Isabel Neatrour, Rhiannon Baggott, Fowzia Ibrahim, Brian Tom, Allan Wailoo, James Galloway, David Scott, Naomi Martin, Richard Jenner, Gabrielle Kingsley, Jonathan Tosh

**Affiliations:** 1 Department of Inflammation Biology, School of Immunology and Microbial Sciences, Faculty of Life Sciences and Medicine, King’s College London, 10 Cutcombe Road, Denmark Hill, London, SE5 9RJ UK; 2 Florence Nightingale Faculty of Nursing, Midwifery and Palliative Care, King’s College London, 57 Waterloo Road, London, SE1 8WA UK

**Keywords:** Acceptability, Complex intervention, Feasibility, Intensive management, Motivational interviewing, Practitioners, Qualitative, Rheumatoid arthritis

## Abstract

**Background:**

The TITRATE trial seeks to test whether intensive management is valuable in achieving disease remission in moderately active rheumatoid arthritis. Intensive management is a complex intervention consisting of: 1) 12 x monthly appointments, 2) tailored ‘treatment support’ based on motivational interviewing techniques, 3) optimised medication (including the opportunity for biologics), 4) provision of a Patient Handbook, and 5) shared treatment planning. This study aims to understand: a) patients’ and practitioners’ views on the feasibility and acceptability of intensive management, and b) patients’ and practitioners’ experience of receiving/providing intensive management.

**Methods:**

A qualitative study, nested within a randomised controlled trial. Participants were patients (*n* = 15) in the intensive management arm of the trial and rheumatology practitioners (*n* = 16) providing the intensive management intervention, from 18/42 clinics across England. Data were collected via semi-structured interviews and analysed using thematic analysis and iterative categorization.

**Results:**

Monthly appointments were largely acceptable to both groups who cited several treatment benefits (e.g. regular review of medication, practitioners built close relationships with patients). Practitioners were ‘fairly confident’ using the motivational interviewing techniques. Learning to pace was the most commonly reported self-management technique that patients and healthcare professionals worked on together, followed by gaining control over pain and fatigue. Practitioners liked having the option to offer biologics to patients with moderate RA. Most patients found the optimised medication (following monthly joint assessment) helpful and side-effects experienced were resolved. Variation existed in the extent to which patients engaged with the Patient Handbook and shared treatment planning, with those who did engage doing so in the early stages.

**Conclusions:**

Feedback from patient participants about the intensive management intervention was positive. They found increased medication helpful. Continuity of care with the same healthcare professional at regular intensive management sessions, and the treatment support provided, were highly rated. Feedback from practitioners indicated that intensive management training is feasible. Evidence from the interviews showed that some practitioners applied motivational interviewing techniques during standard care appointments and they would like the opportunity to address lifestyle issues with patients.

**Electronic supplementary material:**

The online version of this article (10.1186/s41927-019-0057-8) contains supplementary material, which is available to authorized users.

## Background

Rheumatoid arthritis (RA) is a chronic autoimmune inflammatory joint disease, which can cause cartilage and bone damage as well as disability [1]. The main physical symptoms are pain, fatigue, stiffness and swelling of the joints [2]. Patients also experience psycho-social consequences, including social isolation and depression [3, 4].

Patients with RA are sub-divided by their disease activity levels; assessed by the disease activity score for 28 joints (DAS28) with scores which range from 0 to 9 [5]. Treatment choices for RA often depend on disease activity (as measured by the DAS28). Many patients that attend rheumatology clinics have ‘moderate’ disease activity [6] (DAS28 3.2–5.1). This means they have achieved some degree of disease control but are not in remission (DAS28 < 2.6).

Biologic therapies were first studied in patients with active disease (DAS28 > 5.1). The National Institute for Health and Care Excellence (NICE) [7] restrict the use of biologic therapies in moderate disease patients; even though evidence indicates that they are effective in less severe disease states [8]. Currently, recommendations for the management of patients with moderate disease activity are treatment with disease-modifying anti-rheumatic drugs (DMARDs) and steroids, and an annual specialist review [7].

There is some evidence which suggests that patients with moderate RA would try intensive management. A qualitative study [9] examined the views and expectations of patients with moderate RA about intensive management strategies. Monthly appointments were largely acceptable to patients, who focused on a desire to improve their physical symptoms, namely reduced pain and better mobility. Patients who did not think that their treatment controlled their RA were more likely to try intensive management. Some, however, expressed concerns about taking higher doses of medication because of the potential side-effects. There was significant heterogeneity in patients’ interest in educational materials. These findings were supported by earlier qualitative studies [10, 11] which investigated the experiences of patients with active RA who were in receipt of biologics.

Several studies have examined the impact clinicians’ support has on patients with RA. A recent systematic review [12] found that higher levels of trust in the clinician and active patient participation in the medical consultation were linked to lower disease activity, better global health, and more positive beliefs about their control over the disease. A cross-sectional survey [13] with patients diagnosed with inflammatory arthritis revealed that poor communication (e.g. rushed consultations) and clinicians’ reluctance to ask about psychological and social problems, left patients feeling unsupported. Conversely, patients experienced less psychological distress when clinicians helped them to adjust to their life with inflammatory arthritis.

Furthermore, a qualitative study [14] explored rheumatology clinicians’ (including nurses) experiences of training courses designed to facilitate patient self-management. Clinicians appreciated explanations of the theory which underpins self-management. All clinicians identified learning from the continuous professional development training which enhanced their clinical practice, such as the use of tools and techniques to support patients’ behaviour change. Clinicians found it challenging, however, not to problem solve on behalf of their patients. Concerns included how clinicians need to respond if complex psychological issues arose during a consultation. Patients who attended routine consultations with the clinicians who attended the training courses were interviewed [15]. They appreciated the open, patient-centred, communication style and clinicians’ understanding of the different ways in which inflammatory arthritis affected them. Patients seemed to be able to self-manage their long-term condition better when they were actively involved in their own care.

Evidence therefore suggests that intensive management may improve outcomes for patients with moderate RA. The Treatment Intensities and Targets in Rheumatoid Arthritis Therapy (TITRATE) trial seeks to examine whether intensive management strategies improve outcomes for patients with moderately active disease, defined through achieving remission at 12 months. Intensive management involves the combination of treatments including DMARDs, steroids and sometimes biologics, together with a ‘treatment support’ programme of non-drug interventions and psychosocial support [16]. The intensive management intervention was delivered by rheumatology practitioners (who were mostly nurses) across sites in England, full details of the intervention are reported elsewhere [16]. Briefly, the intensive management intervention was a complex intervention [17] that consisted of several interlinked components: 1) 12 x monthly appointments with rheumatology practitioners for up to 1 h, 2) tailored ‘treatment support’ based on motivational interview techniques [18], 3) increased medication according to an agreed treatment algorithm, based on monthly disease activity assessments (which included the opportunity to prescribe biologics), 4) receipt of a Patient Handbook to support intensive management (developed with patients and clinicians) [19], and 5) a ‘shared treatment plan’ (developed with patients).

Qualitative studies which are nested in trials are uncommon, however, they are useful to explore recipients’ and providers’ responses to complex interventions [20]. This study aims to understand: a) patients’ and practitioners’ views on the feasibility and acceptability of TITRATE intensive management, and b) patients’ and practitioners’ experience of receiving/providing TITRATE intensive management.

## Methods

This was a qualitative study nested within the TITRATE trial [21]. We recruited patient and practitioners from 42 rheumatology outpatient clinics across England. There were no additional patient inclusion or exclusion criteria for this study to those of the trial [7] (see Additional file 1). Practitioners were trained healthcare professionals, who were often but not always nurses, identified by the Principal Investigator as being competent to provide the intervention [16]. The inclusion criterion for practitioners for this study were to have delivered at least six intensive management sessions with the same patient. The rationale for this criterion was to allow healthcare professionals time to have applied the intensive management strategies and techniques prior to the semi-structured interview.

### Recruitment

During their first intensive management session, practitioners provided each patient with written information about a ‘sub-study’ which included an optional semi-structured interview. The invitation letter requested that patients who chose to participate in the sub-study should complete the consent form and return the document directly to the lead researcher (LP). Patients who chose to take part in the semi-structured interview were asked to sign a second consent form. Practitioners were sent an e-mail with a participant information sheet attached. Recruitment to the study was stopped once there were no further themes emerging from the data.

### Data collection

The lead researcher (LP) developed the semi-structured topic guides for patients and practitioners (see Additional file 2). These were based on the five constituent components of the intensive management intervention. Questions focused on the acceptability and feasibility of the intensive management intervention. The semi-structured topic guides were discussed with the multi-disciplinary research team and a departmental Patient Expert (SdS) who provided feedback on the suitability and relevance of the questions [22]. Topic guides were also reviewed by the two other researchers (HL, JS).

The lead researcher (LP), who has experience of conducting qualitative research and was not involved in the direct care of any of the patients, carried out the audio-recorded interviews between February 2016 and September 2017. Individual interviews were conducted with patients (*n* = 15) and practitioners (*n* = 13) [23], both face-to-face (*n* = 8) and over the telephone (*n* = 20) [24]. One focus group was also held with three practitioners which used the same topic guide as the interviews. Interviews with patients were arranged for a date after patients had completed all their intensive management sessions, in case the interview influenced their views about the trial intervention. No pilot interviews were held; however, slight adjustments were made to the topic guides following the first two interviews with each group (e.g. the wording of the questions). Ethics approval for the patient sub-study was obtained from West London & GTAC Research Ethics Committee (Reference: 13/LO/1308) on 3rd December 2014. The interviews with the practitioners were considered a service evaluation; therefore, a formal Research Ethics Committee approval was not required.

### Data analysis

Interviews were transcribed verbatim, by an external professional agency, and coded shortly after completion in order to inform when data saturation was reached. A deductive approach to analysis was taken based on the interview schedule [25]. The lead researcher (LP) analysed both sets of transcripts through thematic analysis [26] and iterative categorization [27] (supported by NVivo 10, a qualitative computer software programme). Iterative categorization generates a clear audit trail with the data analysis, closely linked to the raw data and involves four stages: 1) familiarisation through the reading of transcripts, 2) line by line coding to organise the data in preparation for analysis, 3) descriptive analysis which identifies themes, and 4) interpretive analysis that explores patterns, inconsistencies and relates findings to existing knowledge.

To validate the data, a second experienced qualitative researcher (HL) cross-referenced the emergent themes with the lead researcher (LP), and consensus between both researchers was reached. In addition the lead researcher (LP) referred to the original transcripts throughout the analysis, and deviant accounts were included [28].

## Results

Table 1 show the patient demographic information. Fifteen patients (12 females:3 males) from ten rheumatology clinics consented to participate. Patients were all Caucasian, with a mean age of 58 years (range 35–70 years old),

**Table 1:** Patient demographic information

Number	Gender	Age range (years)	Self-reported ethnicity	Site
1	Female	65+	White British	A
2	Female	55–64	White British	A
3	Female	55–64	White British	A
4	Male	65+	White British	B
5	Female	65+	White British	B
6	Female	45–54	White Caucasian	B
7	Female	55–64	White British	C
8	Male	35–44	European White	C
9	Female	65+	White British	D
10	Female	55–64	White British	E
11	Female	65+	White British	F
12	Male	35–44	European White	G
13	Female	65+	White British	H
14	Female	55–64	White British	I
15	Female	35–44	White British	J

Table 2 shows the practitioner demographic information. Sixteen practitioners (13 research nurses, 3 specialist nurses) from 13 rheumatology clinics consented to participate. Practitioners were all female, with a mean age of 49 years (range 30–70 years).

**Table 2:** Practitioner demographic information

Number	Role	Site
1	Research nurse	A
2	Research nurse	A
3	Specialist nurse	A
4	Research nurse	B
5	Research nurse	B
6	Specialist nurse	C
7	Research nurse	D
8	Research nurse	E
9	Research nurse	F
10	Specialist nurse	G
11	Research nurse	H
12	Research nurse	I
13	Research nurse	J
14	Research nurse	K
15	Research nurse	L
16	Research nurse	M

Based on constituent parts of the intensive management intervention, 13 themes were identified (see Table 3).

**Table 3:** Constituent components of the intensive management intervention and identified themes

Components of the intensive management intervention	Themes
1. Monthly Appointments	Monthly appointments acceptable
	Access to services/consultant
	Monthly appointments beneficial
	Intensive management preferable to standard care
2. The Therapeutic Relationship	Practitioners ‘fairly’ confident using motivational interviewing techniques
	Patients and practitioners work on goals together
	Importance of continuity of care
	Provision of helpful information
3. Increased Medication	Improvement in RA symptoms
	Side-effects of medication
	Treatment algorithm easy for practitioners to use
4. Patient Handbook	Views on the content of the Handbook
	Introductory use of Handbook
5. Shared Treatment Plan	Views on the shared treatment planning

### Monthly Appointments

#### Monthly appointments acceptable

Patients did not mind attending the monthly intensive management sessions with the rheumatology practitioners. Facilitators to attend the appointments included patients being retired and their healthcare professionals’ flexibility with the proposed future monthly dates. The main enabler, however, was that patients experienced that the sessions were valuable, because they were ‘getting something from them’. The patients who were employed or had family commitments, e.g. caring for an unwell relative, found the monthly appointments demanding. Despite this, they were pleased to have participated in the trial for the twelve-month duration.

*‘With working full-time as well and having to go up there [clinic appointments], it was difficult, because in work I had to work my breaks to be able to make those appointments. So there was difficulty in getting there [clinic appointments], but I wanted to go.’ (10F55–64)*

Thirteen of the practitioners were research nurses and, in their roles, they come across different pressures. Overall, practitioners successfully managed to see patients for monthly intensive management appointments. Facilitators to successfully manage the monthly consultations were having enough time to space the appointments out and meeting motivated patients who were keen to attend. Problems encountered were mainly logistical (e.g. room availability in the clinic). Some commented that seeing patients every month would be more of a challenge if multiple patients were attending intensive management sessions.

*‘I book the patient in knowing - I'm not booking them in at 10 o'clock, knowing I've got another patient at 11. I book a 10 o'clock slot with a possible other patient at 12 o'clock. So in that respect I'm fortunate.’ (9RN)*

#### Access to services/consultant

One of the main advantages of monthly appointments was increased access to services (e.g. physiotherapy, occupational therapy, neurology, x-rays, MRI scans). Any concerns patients raised during the session, the practitioner acted upon promptly. The additional contact with the consultant rheumatologist (either directly or via their rheumatology practitioner) and the opportunity to receive a steroid injection straight away, were reported as advantageous by patients and practitioners.

*‘There were a few things [medical concerns] I mentioned and immediately the consultant said “Right, well we'll have an MRI scan on that, we'll have x-rays on it”. It was really good stuff.’ (4M65+)*

#### Monthly appointments beneficial

Patients and practitioners found the monthly appointments helpful as medication could be reviewed more regularly, treatment could be changed faster to control the disease, and regular appointments were a ‘reassurance’ to patients if a problem occurred (e.g. side-effects from medication). Monthly appointments allowed for regular blood monitoring.

*‘If they decided that I needed a change in medication or increased the medication [ … ] they [practitioners] were closely monitoring it [patient’s response to medication]. It's like fine-tuning an engine really. That's how I equate with it [process of medication titration].’ (4M65+)*

Patients appreciated the additional time with the practitioner and reported feeling ‘safe’ and ‘looked after’. They described professionals as someone to talk to about their emotional state, with whom they could discuss ideas with and ask for advice.

*‘I found them [sessions] very, very useful definitely. It [attending the sessions] was extremely good in helping me to go forward, understand what was happening with my body, having everything checked out properly, not feeling that you were on your own in a dark tunnel.’ (2F55–64)*

Healthcare professionals enjoyed meeting regularly for the intensive management sessions ‘to establish a proper relationship’, ‘build rapport’ and ‘really get to know’ their patients. They recognised that patients appreciated their support.

*‘I think it's also helpful because prior to them [the patient] coming into the study you don't know the patient so you're building up that rapport all the time and by seeing them more regularly.’ (9RN)*

#### Intensive management preferable to standard care

Those interviewed either saw their consultant annually, every 6 months, or every 3 months under standard care [7]. Problems conveyed because of the infrequent appointments included patients not seeing the consultant when their RA was at its worst and them finding it difficult to recall how they had felt physically since their previous appointment. Consultations were described as ‘rushed’ with little time for discussion.

*‘[ … ] and then you go once a year but you just feel like a number. This is this, this is this, okay, how is it [RA]? Fine. We'll see you in another 12 months and you're just left like oh my God.’ (15F35–44)*

Practitioners reported routine appointments as too medically focused, with a limited time to discuss change of lifestyle factors. Due to seeing more patients for shorter appointments, they tended to review patients and check their disease activity only. Several interviewees mentioned they prefer the care patients receive in the intensive management arm and think it is unfortunate there is not the time to deliver the same level of care in standard practice.

*‘If you've got a clinic of seven [patients booked], you've got to churn them through [ … ] the nurse being a pastoral carer has gone. We're basically following up and checking their disease activity scores and things like that now [ … ] Whilst you get that pastoral care with the TITRATE and they [patients] see it [pastoral care], they love it.’ (1RN)*

### The Therapeutic Relationship

#### Practitioners ‘fairly’ confident using motivational interviewing techniques

Practitioners’ confidence to deliver the motivational interviewing techniques varied between individuals, however, most described themselves as ‘fairly confident’. They explained how the techniques seemed to be difficult at the beginning of the application but became easier with practice. Some practiced the motivational interviewing techniques during standard care appointments. Facilitators to delivery included to meet several intensive management patients close together, who responded and progressed well, and previous experience of delivering psychoeducational interventions.

*‘I think that they [the sessions] become easier as the sessions develop. I think just that first two or three [sessions] when you don’t really know each other and you’re trying to encourage that - to encourage the conversation more than just a yes or a no, it’s quite difficult.’ (7RN)*

*‘I'm probably fairly confident because I've done this counselling course in the past.’ (5RN)*

Several interviewees explained the difficulty to change their communication style towards a patient-centred approach. Reasons provided were that they had ‘spent so long doing consultations a certain way’ and the newly acquired approach being different. Specific challenges reported by practitioners included the use of open-ended questions, letting patients talk, and not telling patients what to do.

*‘That's the bit that I find really hard with motivational interviewing, because they've [patient] probably got about a 10 second span to answer that question, if they [patient] don't, I'm going to fill it in [answer the question] for them.’ (1RN)*

*‘I think that's the thing [motivational interviewing technique] I find the most difficult, is trying to do it [ask a question] in a way that it is an open question, rather than just a yes or no answer.’ (10SN)*

#### Patients and practitioners worked on goals together

Weight loss (either through a change in diet or increased physical activity) and exercise were the most commonly reported goals by patient. Other areas of support included management of fatigue, pain, and regulation of sleep patterns. Patients described how they identified these with their practitioner, who ‘helped to organise them’ and ‘gently encouraged them’ to introduce changes. Practitioners’ monitoring progress at subsequent intensive management sessions was described as helpful, as was having someone to go back to when a plan had not worked, or when they had an idea which they needed to discuss.

*‘I think the combination, the diet with the medicine, it's given me a good effect.’ (12M35–44)*

*‘A couple of the aims and goals I had, like reducing alcohol and doing more exercise, I did do. So it was useful talking to somebody every month.’ (13F65+)*

Learning to self-manage was an important part of the intensive management sessions. Most patients discussed strategies they used to ‘pace’ their activities so as not to get fatigued. These included to break tasks down into smaller parts, recognise when a job is beyond their capability, listen to their body rather than ‘push through’, and not become angry when they cannot do everything they want to. One patient participant reported that the intensive management sessions had changed little about the way she manages her RA.

*‘She [rheumatology practitioner] said try not to do everything all at once [ … ] Daft things like say painting the bedroom, I could have done that in one day at one time, but it's taking us three days now, but it's done.’ (14F55–64)*

*‘I've got to admit I can't really say it has had a lot of impact on my management of it [RA]’ (10F55–64)*

Many healthcare professionals reported that the intensive management sessions had a positive impact on patients’ self-management. Self-management support was less helpful to patients who were already motivated and able to self-manage.

*‘It's a difficult one to gauge because the two patients that are in the intensive management arm are fairly motivated individuals anyway.’ (16RN)*

Practitioners explained how the treatment support had helped some patients to become more self-aware and increased patients’ knowledge of how best to help themselves (e.g. self-management strategies for coping with pain) which resulted in them taking control of their long-term condition.

*‘It’s looking at the bigger picture of what else you can do. It might be that the pain has flared up because they’re [patient] sitting in a chair all day. Or is their mood affected because they’re isolated at home because they’re not able to get out and about?’ (7RN)*

#### Importance of continuity of care

Continuity of care was very important to patients because they could see a practitioner who was already familiar with their medical history/joints, and not have to repeat the same information to a new clinician every visit. Emotional benefits included the participant and practitioner ‘getting to know’ each other. This continuity meant that the patient felt ‘comfortable’ and developed trust and a bond with the practitioner.

*‘After a while once I got to know them [practitioners] we got on a lot better. I was less embarrassed I suppose is the word or maybe less reserved. I was able to talk to them [practitioners] about anything really. I suppose it's building up trust isn’t it?’ (10F55–64)*

#### Provision of helpful information

Most patients said that their practitioner helped them to learn about RA and understand the condition more. Knowledge about RA and its treatments, and what to expect from these, is something patients found valuable. Areas mentioned specifically by patients included fatigue, pain, medication management and the difference between RA and osteoarthritis.

*‘You know I'm just feeling pretty good, because my nurse helped me to understand my illness and she explained clearly how it [the medication] works, what I can expect, and that was a very good experience for me.’ (12M35–44)*

### Increased Medication

#### Improvement in RA symptoms

Most patients found the change in medication, including the use of biologic therapies, helpful. They reported that their RA had improved, and that the symptoms were more controlled. Patients described the impact of this improvement (e.g. improved dexterity, increased physical activity, better self-confidence, return to employment). Steroid injections were regarded as particularly effective for the fast relief of localised pain. A small number of patients felt less satisfied with their treatment e.g. because it was not as effective as their previous medication, and continuous symptoms of RA (such as fatigue and pain). One participant was prevented from starting biologic treatment due to an infection.

*‘I've had arthritis for 34, 35 years [ … ] and only just recently I feel that it's [RA] finally been controlled [ … ] I've got stiff swollen joints that have been damaged, they'll never be repaired I know that, but the fact that I'm not having flare after flare after flare, which I was having that's a great relief to me.’ (1F65+)*

*‘Unfortunately, the first one [drug] they put me on just hasn't worked. I've been having very severe ‘flare ups’ of arthritis. I'm on another one [medication], a biologic now, but unfortunately I've had several infections which haven't allowed me to take it.’ (13F65+)*

#### Side-effects of medication

About half of patients experienced no side-effects during the intensive management intervention. Side-effects encountered by others in the group included steroid-induced weight gain, rash, hair loss and nausea. These were mainly occurred after an alteration of their medication. Most reported that the side-effects subsided or improved with time or the clinician discontinued or modified the drug that caused the side-effect. Nobody reported problems in relation to changes of drug administration (e.g. self-injection) or the delivery of drugs to their home.

*‘At first, I found it very hard, because I was just taking medicines and medicines. I had a few little side-effects - upset stomach and feeling a bit down and drowsy. But overall when I got through the first few weeks, fine.’ (2F55–64)*

#### Treatment algorithm easy for practitioners to use

Practitioners described the treatment algorithm as clear and easy to follow. They stated that the pathway with regards to drug treatment was close to standard care. Research practitioners, who are unable to prescribe, would seek the advice of the consultant before they altered treatment. Some commented that relying on the consultants’ decision was a barrier to the sessions. Others enjoyed the opportunity to learn more about RA medications. Practitioners liked the option to offer biologics to patients with moderate RA, which would not be allowed in routine practice.

*‘The rest of the intensive management stuff I'm absolutely happy with. It's the changes in medication, where I have to rely on other people, to do prescriptions and things like that that I think held me back.’ (12RN)*

### Patient Handbook

#### Views on the content of the handbook

Most patients described the Handbook as ‘useful’, ‘informative’, ‘interesting’ or as ‘a good introduction to the trial’. Some patients, however, did not find the Handbook beneficial. Reasons cited included the content was irrelevant to them, the Handbook presented information in a negative way, and they already knew a lot of the information presented in the document (e.g. to establish a regular sleep pattern, eat five portions of fruit and vegetables per day). Feedback about the activity form and action plans included in the Patient Handbook was also mixed. One person commented that it was more convenient to use the diary on his mobile phone as an activity record rather than writing on the paper copies.

*‘Oh yeah [ … ] it [handbook] was very informative you know, like loads of information and lots of help.’ (14F55–64)*

*‘A lot of it [content of the handbook] wasn't relevant to me.’ (4M65+)*

Feedback from practitioners about the content of the Handbook was very positive. They described the resource as ‘comprehensive’, ‘useful’, ‘clearly written’ and one which patients in the intensive management arm of the trial were pleased to read in their own time. The Handbook provided an important asset for healthcare professionals to refer patients to for help with difficulties such as fatigue and exercise.

*‘I do refer patients back to the information in the handbook when they're, for example, struggling with fatigue or exercise [ … ] I've also given the handbook out or I've shown other practitioners the handbook because it's a great resource.’ (6SN)*

Practitioners utilized the Health Behaviour Check form, Balance Sheets, and Activity Diaries in the back of the Handbook as part of the motivational interviewing approach. They suggested to keep these sheets separate from the rest of the Handbook. In addition, they suggested that the pages could go into a smaller booklet which could be transported more easily and discreetly than the Handbook.

#### Introductory use of the handbook

Despite the mainly positive feedback from most patients about the Handbook, one interviewee did not use the resource and over half admitted that they read the document only at the start of the trial.

*‘I can't say I used it [handbook] often, but I did use it [ … ] I did use it to begin with more than I did at the end.’ (10F55–64)*

Practitioners described two categories of patient: one who would read the Handbook carefully and bring the resource to the sessions, and others who did not show much interest in the material. Although some stated that the patients made use of the resource, the majority explained that patients did not bring the document to their sessions. Practitioners found it, therefore, difficult to engage with patients who were less keen on the content.

*‘We've got those that want to read everything and then you get those that don't want to know anything, just give me a new tablet [pill] and I'll start it.’ (3SN)*

### Shared Treatment Plan

#### Views on the shared treatment planning

Several patients forgot to complete the Shared Treatment Plan with their practitioner. Those who remembered described the Shared Treatment Plan as ‘interesting’, ‘good and comprehensive’ and the process of shared completion as ‘useful’. A few patients thought drafting the Shared Treatment Plan facilitated collaboration between themselves and their practitioner at the start of the trial.

*It [the process of completion] was useful for me to sit down and put it [previous medication, medication preferences] in black and white. I suppose it [the process of completion] was good for him to find out where my head was at.’ (15F35–44)*

Feedback from health care professionals about the Shared Treatment Plan was mixed. Out of those who had completed it, some explained that this process was already standard practice. Others fed back that they had completed the document but did not refer to the plan in later sessions. Positive comments about the shared treatment plan from practitioners revealed that the Plan was helpful when ‘getting to know’ a patient and ‘triggered discussion and more questions’. In addition, they explained in future negotiations about patients’ preference (e.g. unlikely to take steroids) the document was beneficial. Similar to the goal-setting forms, practitioners reported that the Shared Treatment Plan needs to be separate from the Handbook.

*‘Yes [ … ] I have found that [the Shared Treatment Plan] useful but [ … ] I'll be honest I haven't really referred back to it very often. I don't know if that's just a fault of mine or whether it's because the sessions have taken their own path.’ (13RN)*

## Discussion

### Main findings

Consistent with previous research [9] the intensity of monthly appointment intervals over 12 months was acceptable and highly valued by the majority of patients, and also by the practitioners who delivered the intensive management intervention. Practitioners were reasonably confident with the use of motivational interviewing techniques. As observed in previous studies [14, 29] some described difficulty with altering their established consultation style. Learning to pace was the most commonly reported self-management technique which patients and practitioners worked on together, followed by gaining control over pain and fatigue. Patients appreciated the verbal encouragement they received from their practitioner. Consultation with the same healthcare professional for each session had both physical and emotional benefits for patients. Treatment support was helpful for patients who required guidance on how to manage the disease and increased some patients’ self-awareness and sense of control over their symptoms. Practitioners liked the option to offer patients with moderate RA biologics. Most patients found the increased medication helpful, and side-effects experienced by participants were resolved in time. Variation existed in the degree to which patients engaged with the Patient Handbook and Shared Treatment Plan [9, 10]. Both practitioners and patient participants reported a key advantage of the monthly consultations was to acquire more knowledge about their RA and discuss its management.

### Findings compared to wider literature

Patients with RA live with complex symptoms, such as fatigue and pain, which can have emotional, cognitive and behavioural impacts [30]. It is useful to compare our intensive management intervention with those across other diseases, which aim to improve complex symptoms. The British Pain Society has published guidelines for interdisciplinary pain management programmes (PMPs) [31]. Unlike the intensive management intervention PMPs are based on cognitive behavioural principles. There are three different types of programmes: targeted early PMP interventions, standard PMPs (12 x half day sessions), and intensive PMPs (e.g. 15–20 full days). The programmes include education, guided practice on exercise and activity management, goal-setting, identifying and changing unhelpful beliefs and ways of thinking, relaxation and changing habits which contribute to disability. Compared with no treatment or treatment as usual, there is evidence PMPs improve pain experience, mood, coping, negative outlook on pain and activity levels [26]. Qualitative findings from our study highlight that intensive management in RA improved weight loss, exercise, control over pain, and pacing. Therefore, the outcomes are similar to PMPs. It is recommended that self-management principles are introduced early in the pain experience. Early intervention directed at high-risk patients has been shown to be both clinically and cost-effective [30].

For fatigue, physical activity and psychosocial interventions are effective for patients with RA [32]. Moderate physical activity is also recommended to improve cancer-related fatigue as are pharmacological approaches, adjustment strategies (e.g. prioritizing activities, managing stress and anxiety), complementary therapies, and psychological and educational interventions [33]. A review of educational interventions for the management of cancer-related fatigue in adults showed they may have a small effect on reducing fatigue (its intensity, interference with daily life, general/overall fatigue) and could have a moderate effect on decreasing fatigue distress [34]. Due to the complexity of this symptom, the review authors concluded that educational intervention would result in greater reductions in fatigue when employed in conjunction with other interventions [34]. This supports the complex design of our intensive management intervention, which utilizes educational materials, plus pharmacological and psychosocial intervention.

### Strengths and limitations

This study has provided detailed insight into patients’ and practitioners’ views of the intensive management intervention within the context of a pragmatic trial. As far as we are aware, this is the first qualitative study to evaluate the experiences of patients with moderate RA that receive intensive management. Recruitment of patient and practitioner participants from a total of 18/42 diverse geographical sites across England was a strength as the experiences of clinicians and patients from different clinics were evaluated [35].

Limitations of the study include the use of a potentially biased sample. Both practitioners and patients volunteered to take part in the interviews. It is possible that those who engaged more with the delivery or receipt of the intensive management intervention would be keener to participate. Patients were largely elderly, retired, and all Caucasian, therefore, not representative of the RA population. Practitioners were mostly research nurses (13/16). Research nurses face different barriers compared to specialist nurses (e.g. relying on consultants to change patients’ medication, less experience of regularly seeing patients with RA). Feedback from practitioners in relation to the intensive management intervention may have been different from clinicians delivering standard care. The ratio of research nurses to specialist nurses in the interviews was, however, broadly proportional to the number of research nurses/specialist nurses who delivered the intensive management sessions in the trial.

### Recommendations for future research

Overall, the intensive management intervention was acceptable to patients, whose feedback was positive. Participants found the increased medication helpful, however, seeing the same practitioner at regular intensive management sessions, and the treatment support they provided, were equally as significant.

There is evidence from these interviews that practitioners would like the opportunity to address lifestyle issues with patients in routine appointments. The restricted length and frequency of standard care appointments is an important limitation to quality care provision. Some practitioners did, however, report using the motivational interviewing techniques with patients they see in the standard care pathway. This suggests practitioners were able to transfer some of the skills they learned during the training and use them in time-limited appointments. Feedback from practitioners also indicates that intensive management training is feasible. Future research could examine the most practical and cost-effective way to deliver both this teaching to practitioners, and, the intensive management components to patients with moderate RA in standard care.

## Conclusions

Both patients’ and practitioners’ feedback about the feasibility and acceptability of the intensive management intervention was positive. Patients found the increased medication and support from the practitioner helpful. Practitioners enjoyed taking a more holistic approach, which was facilitated by the longer sessions. These findings indicate that patients with RA would like more continuity of care and support with symptom management.

## Additional files

Additional file 1: TITRATE trial patient inclusion/exclusion criteria. (DOCX 18 kb)

Additional file 2: Patient/practitioner topic guides. (DOCX 24 kb)

## Abbreviations

DAS28: Disease activity score for 28 joints
DMARD: Disease modifying anti-rheumatic drug
PMPs: Pain management programmes
RA: Rheumatoid arthritis
TITRATE: Treatment intensities and targets in rheumatoid arthritis therapy
