# Supplementary material for: Intensive management for moderate rheumatoid arthritis: a qualitative study of patients’ and practitioners’ views
Source: BMC Rheumatol. 2019 Mar 28;3:12. doi: 10.1186/s41927-019-0057-8 (PMC6437952; doi:10.1186/s41927-019-0057-8)
Supplement: Supplementary file 1 — TITRATE trial patient inclusion/exclusion criteria. (DOCX 18 kb) [file 41927_2019_57_MOESM1_ESM.docx]

**TITRATE trial patient inclusion/exclusion criteria**

**Inclusion criteria**

1. Men and women aged over 18 years
2. Diagnosis of RA (by American College of Rheumatology (ACR), 2010 criteria) (1)
3. Have received at least one DMARD for at least six months, and currently receiving at least one DMARD
4. Have intermediate disease activity, defined by: (a) DAS28-ESR 3.2-5.1; (b) at least three active joints (defined as swollen and/or tender) on 66/68 joint count, to include at least one swollen joint
5. Willing and able to follow an Intensive Management Programme
6. Able and willing to give informed consent

**Exclusion criteria**

1. Major co-morbidities making intensive treatment inadvisable (e.g. heart failure)
2. Previously failed multiple DMARDs (at least five treatments) or having received biologics
3. Irreversible disability from extensive joint damage (e.g. replacement of three or more major joints)
4. Women who are pregnant, breast-feeding or at risk of conceiving
5. Current or recent (within the 12 weeks prior to randomisation) participation in another interventional trial
6. Currently in an early RA pathway, which is a 12-month treatment programme for patients with early inflammatory arthritis, in which patients receive intensive treatment with DMARD combinations and steroids and are reviewed monthly by specialist nurses or equivalent members of the rheumatology team; this is one of the current NHS Best Practice Tariffs (2)
7. Aletaha D, Neogi T, Silman AJ, Funovits J, Felson DT, Bingham 3rd CO, et al. 2010 rheumatoid arthritis classification criteria: an American College of Rheumatology/European League Against Rheumatism collaborative initiative. Ann Rheum Dis. 2010;69:1580–8.
8. 2016/17 National Tariff Payment System. Monitor (March 2016) Publication code: IRG10/16.
