# Supplementary material for: Intensive management for moderate rheumatoid arthritis: a qualitative study of patients’ and practitioners’ views
Source: BMC Rheumatol. 2019 Mar 28;3:12. doi: 10.1186/s41927-019-0057-8 (PMC6437952; doi:10.1186/s41927-019-0057-8)
Supplement: Supplementary file 2 — Patient/practitioner topic guides. (DOCX 24 kb) [file 41927_2019_57_MOESM2_ESM.docx]

**Patient Topic Guide**

1. **Tell me what you think about the intensive management sessions?**

-What was it you liked/didn’t like in particular?

1. **I’d really like to hear your thoughts on different aspects of the intensive management:**
2. What are your thoughts on the **Patient Handbook?**

-How often did you use it?

-If not often, why?

**OR**

-What in particular was useful/not useful?

-Was anything missing?

-Do you think you will use the Patient Handbook now the sessions have finished?

-What do you think you will use it for? (E.g. as a reference/activity diaries)

b) What was your experience of completing the **shared treatment plan** with your rheumatology practitioner?

c) How did you find the **increased medication** over the course of the sessions?

d) How did you find going for **monthly appointments**?

1. **How was your interaction with the rheumatology practitioner?**
2. **How supported did you feel?**

-If felt supported, how did the practitioners know how to best support you?

1. **Tell me about how your RA is now compared to before starting the trial?**
2. **Has there been any impact upon the way you manage your RA?**

-How?

1. **To what extent would you say this change has impacted the quality of your life?**

*[Prompt – fatigue, mobility, social life, relationships-have others noticed a difference?]*

1. **How do you anticipate self-managing your RA and coping in the future?**
2. **I’d really like to hear your thoughts about anything you would change about the intensive management sessions?**
3. **Do you have any other feedback?**

**Practitioner Topic Guide**

1. **Tell me what you thought about the intensive management training?**

-What was it you liked/didn’t like in particular?

1. **a) What aspects of the intensive management training were most useful for you?**

-Why

1. **What aspects of the intensive management training were least useful for you?**

-Why

*[Prompt - 2 day training, training manual, supervision calls after first three sessions, webinars]*

**c) What would you change to improve the training?**

-Can you give an example of what might have helped?

*[Prompt - content, duration of training, teaching methods]*

1. **What are your thoughts on the Patient Handbook?**

–Can you give an example of what a patient did or said?

–Can you give an example of how you referred to it or learnt from it?

1. **Can you think of a recent patient where you used the shared treatment plan, can you describe this for me?**

-How typical or different was this patient from others in the study?

1. **I’d like to ask about the treatment algorithm now…**

–Generally how did you find it?

–Can you give me an example of when it was really useful with a patient?

–How about when it was less useful or the patient was unhappy to follow it, can you tell me about that?

1. **How did you find seeing patients for monthly appointments?**

-How manageable was this for you?

-How did it impact the patients?

1. **What experience do you have previously of engaging with patients in this more intensive way?**
2. **What do you think about motivational interviewing?**
3. a) How do you feel about using the **motivational interviewing** techniques?

b) Tell me about the motivational interviewing techniques that you find easier to use?

1. Tell me about the motivational interviewing techniques which you find more difficult to use?
2. How applicable do you feel motivational interviewing is to your future clinical practice?
3. **What is your impression of how the sessions have impacted upon your patients’ self-management?**

-if benefitted, exactly how do the sessions help them? (I.e. what aspects have impacted them?)

1. **I’d really like to hear your thoughts on anything you would change about the intensive management sessions?**
2. **Do you have any other feedback?**
